# Current Development Status of Forest Therapy in China

**DOI:** 10.3390/healthcare8010061

**Published:** 2020-03-17

**Authors:** Zhiyong Zhang, Peng Wang, Yue Gao, Bing Ye

**Affiliations:** Research Institute of Forestry Policy and Information, Chinese Academy of Forestry, Beijing 100091, China; zzy100083@163.com (Z.Z.); wpeng.up@foxmail.com (P.W.); ziguang_yue@163.com (Y.G.)

**Keywords:** forest therapy, definition, public health, forest wellness, challenges, policy suggestions

## Abstract

As a result of rapid urbanization and urban sprawl, natural ecosystems are shrinking or are fragmented, affecting people’s health and quality of life. Modern people prefer to live in large cities rather than rural areas because of greater convenience and more comfortable living conditions. As a consequence, people are suffering from many psycho-physiological health problems and have a longing for natural environments to escape the concrete jungle. Forest therapy has emerged as a preventive and alternative therapy to cope with stress and enhance people’s health and wellbeing as a result of spending time in a green and healthy environment. Here, we review the activities related to forest therapy in China and discuss the commonalities and differences between the forest therapy types. Furthermore, we summarize the current achievements of forest therapy in basic research and the development of the forest therapy industry. We also describe the challenges that forest therapy has been facing. Finally, we provide suggestions for further development in research and industry.

As a result of rapid urbanization and urban sprawl, natural ecosystems, such as forests, green spaces, and water bodies, are adversely affected, are shrinking, or are fragmented [1,2,3], leading to changes in the structure and function of natural systems near urban areas [4,5,6]. Environmental pollution, food security, and work pressure influence people’s health and quality of life [7,8]. As people are becoming increasingly aware of their psycho-physiological health, they have a desire to escape the concrete jungle and long for a natural environment. 

Forests have always played an important role on satisfying the demand for natural environments. Research has demonstrated that a forest environment has positive influences on human health [9,10,11]. When people are walking in forests or green spaces, nature helps them to regain attention and focus, improve their psychological state, and feel free and relaxed [12,13,14]. Recent medical studies have shown that forest environments provide benefits, such as lowering blood pressure, pulse rate, and sympathetic nerve activity, decreasing salivary cortisol concentrations of stress hormones, and increasing natural killer (NK) cell activity [7,15,16,17]. Therefore, forests are being viewed increasingly as being beneficial to people’s health rather than only providing timber. 

Philosophical theory about the relationship between human and nature have existed since ancient times in China. People using plant and nature as a medical treatment to promote their psycho-physiological health have been highlighted. The Taoist Culture of Pre-Qin Dynasty is one of the essences of Chinese traditional culture, containing the ecological wisdom of the relationship between human health and nature [18]. The philosopher Lao Zi encouraged people to learn from nature, Zhuang Zi believes that nature is the mother of all things. They advised people to integrate their mind into nature, achieved sublimate spiritual comfort, and got the harmony between humans and nature. In traditional Chinese medicine, plants and forests have a long tradition of being used as medicine materials or backdrop to promote human health, the health preserving theory of “Nourishing yang in spring and summer, while nourishing yin in autumn and winter” has been widely used since the beginning of Yellow Emperor’ Internal Classic [19].

In recent years, over 30 percent of adults suffer from obesity, diabetes, hypertension, and other lifestyle diseases in China [20]. Driven by evidence-based research, people pay attention to the prevention and treatment effect of forests on lifestyle diseases. Forest therapy has emerged as a preventive and alternative therapy to cope with stress and enhances people’s health and wellbeing by way of spending time in a green and healthy environment [15]. However, in China, forest therapy has not been clearly defined to date, and forest therapy activities are often confused with other green fitness activities. Therefore, we review different types of forest therapy in China, describe their current status, challenges, and suggestions for further development. 

## 1. Definitions of Forest Therapy

Forest therapy activities in China include forest rehabilitation and recreation, forest healthcare, forest tourism, forest experiences, and forest wellness. The commonalities among these activities are the forest environment, which provides the setting, and the goal of health promotion, whereas the differences are the paths used to achieve the goal. 

### 1.1. Forest Rehabilitation and Recreation

Different government departments and scholars have defined forest rehabilitation and recreation in different ways based on their understanding and perspectives. Wu et al. [21] defined forest rehabilitation and recreation in a broad and narrow sense, respectively. In a narrow sense, it is defined as activities occurring in a high-quality forest environment benefitting people’s physical and mental health. This activities of forest rehabilitation and recreation is based on existing health theories and is supported by traditional and modern medicine, including forest healthcare, rehabilitation, recovery, health maintenance, wellness, as well as recreation, travel, and outings. In a broad sense, it refers to all activities to maintain, sustain, and restore human health occurring in a forest environment. 

In March 2019, the State Forestry and Grassland Administration (SFGA), the Ministry of Civil Affairs (MCA), the National Health Commission (NHC), and the State Administration of Traditional Chinese Medicine (SATCM) jointly published the Opinions on Promoting the Development of Forest rehabilitation and recreation Industry. This report defined forest rehabilitation and recreation as a series of service activities conducted in the forest environment for the purpose of promoting public health, the forest resources, landscape resources, food and drug resources, and cultural resources were utilized and these activities integrated with medicine and health care science for health care, rehabilitation, and elderly care. 

In summary, the important words in the definitions are “quality forest + medical and health care + physical and mental health”, meanwhile different types of activities are used for enhancing health, such as sports, entertainment, and outings. 

### 1.2. Forest Healthcare

There is no clear and consistent definition of forest healthcare in China, but the following elements are included: (1) forest medicine as the core and evidence-based medical research as the foundation; (2) conducted in a forest environment; (3) focused on disease prevention, stress relief, and health promotion [21,22]. 

Considering these elements, we believe that forest healthcare represents a sound approach to prevent and cure disease in a forest environment. When forest healthcare is used as a preventive therapy, the health-promoting functions of forests have been proven by medical experiments, and the activities of forest healthcare are conducted under the guidance of doctors or therapists. More importantly, the forest environment for healthcare has two objectives: to stimulate the five senses of humans and have a positive effect on the human body.

### 1.3. Forest Tourism

Forest tourism is a type of eco-tourism and popular recreational activity in China because it meets people’s needs for a green and healthy life. Forest tourism has become an important pillar of the tertiary industry and refers to tourism activities in a forest landscape [23]. The landscape resources include not only animal and plant resources but also the eco-environment and cultural resources [24]. 

Forest tourism is a traditional approach for the use of forest resources. In short, it refers to any form of tourism activities in forests, either in a forest environment or by using forests as a backdrop [25]. Therefore, forest tourism can also be defined in a broad or narrow sense. Many people choose to walk, recreate, and have cook-outs in forests. In general, people regard forest tourism as an opportunity to get close to nature, and focus more on visual experiences [24]. 

### 1.4. Forest Experience

In China, forest experience is seen as an opportunity to understand nature. It expands forest tourism towards a participatory and interactive activity and gets people closer to forests. 

Forest experience has been used as the general term for people’s activities focused on perceiving the forest and its environment through various senses in the Notice on Promoting Forest Experience and Forest Wellness Development proposed by SFGA (the former State Forestry Administration, SFA). Cheng et al. [26] described forest experience as a practice of using forest resources and forest landscape and guiding people to sense and understand the relationship between forest and human through sensual experience, thereby promoting physical and mental health and inspiring people to protect forests actively to achieve sustainable forest development. In forest experience, the infrastructures are built to enable recreation and enjoyment in forests, and the facilities and oral introductions are provided as a guide of exposing to forests and experiencing their beauty. Zhang et al. [27] categorized forest experiences into sightseeing, cognitive, and recreational experience. 

Forest experience emphasizes the interaction between humans and nature, the collective participation of target groups, and the satisfaction of emotional needs [28]. 

### 1.5. Forest Wellness

Forest wellness is an emerging activity that combines human wellness and forest environment and was first proposed by SFGA in the Notice on Promoting Forest Experience and Forest Wellness Development. Forest wellness is based on a high-quality forest environment and green forest products, and it refers to all activities that improve people’s health and prevent, relieve, and cure diseases [23,29]. In simple words, forest wellness refers to forest-based activities to maintain health. 

Forest wellness is often combined with the traditional Chinese medicine [30], and mineral hot springs in forests. Health maintenance programs have been developed to prevent disease and enhance psycho-physiological health [29]. 

**Figure 1:**
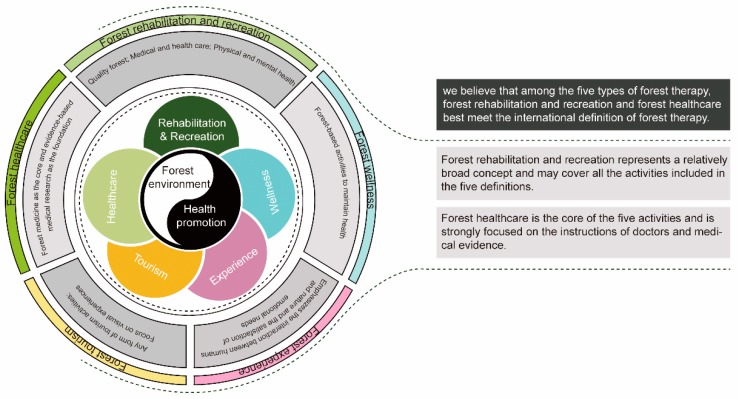
Relationship between the five types of forest therapy.

Considering the current progress of forest therapy in China, we believe that among the five types of forest therapy, forest rehabilitation and recreation and forest healthcare best meet the international definition of forest therapy. Forest rehabilitation and recreation represents a relatively broad concept and may cover all the activities included in the five definitions. However, forest healthcare is the core of the five activities and is strongly focused on the instructions of doctors and medical evidence (Figure 1).

## 2. Development and Current Status

After researching German forest therapy and Japanese Shinrin-yoku, in the 1980s, the Forestry Bureau of the Agriculture and Forestry Department of Taiwan, China and the researcher Liu published the books Forest Bathing—The Latest Fitness and Forest Bathing—Green Fitness in March and June of 1984, respectively. The two books systematically introduced the type, content, approach, case studies, and research of forest therapy in Germany, Japan, and the UK [31,32]. Subsequently, the concept of forest therapy was introduced, and a green fitness fashion swept over Taiwan, China. 

In mainland China, the Foreign Project Cooperation Center (FPCC) of SFGA and the Beijing Municipal Bureau of Landscape and Forestry (BMBLF) introduced the Japanese concept and model of forest therapy in 2012. The BMBLF sponsored the translation and publishing of Li’s Forest Medicine (in Chinese) in 2013, which began the promotion and practice of forest therapy in mainland China. Today, significant achievements have been made in basic research and industry of forest therapy. 

### 2.1. Basic Research

After the concept of Japanese forest therapy was introduced by the FPCC and BMBLF, researchers in China began to study the relationship between forest therapy and human health. Mao et al. [33] selected a group of male students (average age of 20.79 ± 0.54) as subjects of a 2-night forest therapy session and found that mid- and short-time exposure to an evergreen broad-leaved forest reduced oxidative stress and pro-inflammatory and serum cortisol levels. After seven days and nights under controlled test conditions in the forest, the patients’ blood pressure was significantly lowered, and negative emotions were reduced [34]. A pilot study in Guiyang found that exposure to forests relieved anxieties related to financial difficulties, exam pressure, and relationships [35]. Another study showed urban park scenes relieved stress and restored attention levels, whereas viewing urban roadways increased negative feelings [36]. In another study, elderly patients with chronic heart failure (CHF) who had experienced the first forest bathing trip were again recruited to a second 4-day forest bathing trip after 4 weeks. The study showed that a steady decline in the brain natriuretic peptide levels, and an attenuated inflammatory response as well as oxidative stress. The additive benefits of twice forest bathing trips in elderly patients with CHF were demonstrated [37]. Guan et al. [38] have investigated the effects of different tree species on anxiety alleviation. University students were recruited to visit urban forests dominated by birch (*Bet¬ula platyphylla* Suk.), maple (*Acer triflorum* Komarov), and oak (*Quercus mongolica* Fisch. ex Ledeb) trees. The results showed that the anxiety of the participants was reduced in the maple forest, the largest anxiety alleviation effects were observed in the birch forest, and female participants perceived more anxiety alleviation than male participants. This study provides information on the relationship between forest species and human health factors and guides the direction of future research. 

From physiological and psychological perspectives, the evidence-based research findings pointed to a reduction in human oxidative stress, serum cortisol levels and blood pressure and an increase in relaxation for participants exposed to forests. Some studies involving the use of videos of nature had also confirmed the same psychological effects. In general, most studies, conducted by medical personnel, recruited the patients that are diagnosed with essential diseases as the participants. The methods of detecting biochemical indicators were used to quantitatively compare the changes before and after spending in the forest. The studies of researchers majoring in forestry and landscape architecture mainly recruited the healthy, young university students as the participants. Questionnaire surveys were often provided to compare the differences between different scenes, which was difficult to represent the physiological indicators of human body. Gratifyingly, due to more familiarity with the forest environment, they devoted themselves to compare the differences between the effects on humans of different green environments, such as forests, water bodies, and other green spaces. 

The study methods were mainly comparative experiments, most of which take the urban environment as the control, the number of participants ranged from 20 to more than 200, and the experiment cycle is 2–7 days, as explicated within the key in Table 1. 

The results of these studies were consistent with the conclusions of studies by Shin [43], Chun [44], and Ochiai [45] in Japan, Korea, and in the world. However, medical evidence-based studies on the effects of forests on human health in China are scarce and should be the focus of future studies. In China, researchers have focused primarily on the ecological health factors in the forest environment, e.g., the differences in ecological factors between forest and city environments [46], the dynamic changes in the comfort index of forests, volatile organic compounds (VOCs) of plants, negative oxygen ions [47,48,49,50,51], as well as the impacts of VOCs from branches and leaves of conifer species on the locomotor activities of mice [52]. The differences in the ecological health factors of different tree species were also investigated [53,54]. On this basis, the studies on the relationship between forest environment and human health should be carried out in the future.

### 2.2. Development of the Forest Therapy Industry

Health issues are a public concern. Since the national strategy “Healthy China” was launched, forest therapy, which is an integral part of the health industry, has developed rapidly in China [21]. 

In recent years, a series of policies that guide and promote the forest therapy industry have been implemented, including the Opinions of the State Council on Promoting the Health Service Industry, the Opinions of the State Council on Accelerating the Development of the Elderly Care Service Industry in 2013, the Standards for National Demonstration Base of Rehabilitation and Recreation Tourism in January 2016, the Notice of SFA on Promoting Forest Experience and Forest Wellness by the Station of Forest Farm and Nursery (SFFN) of SFA in January 2016, the Notice on the Pilot Program of the Forest Experience Base and Forest Wellness Base by the Forest Park Management Office (FPMO) of SFA in February 2016, the Outline of Ecological Culture Development in China (2016--2020), The 13th Five-year Plan for Forestry Development, and the Outline of the “Healthy China 2030” Plan. These policies provide clear guidance on the development of the forest therapy industry. 

Multiple institutions and organizations dedicated to the development of forest therapy were founded after the implementation of the policies. On 18 September 2015, the Forest Medicine and Health Promotion Committee (FMHPC) of the China Forestry Industry Association (CFIA) was founded, with the goals of connecting different industries, improving forest medicine and health industry, and promoting coordinated and sustainable development of forest medicine, health care, recreation, and wellness. On 14 October 2015, the International Forest Therapy Cooperation Committee (IFTCC) of the China Forestry Economy Society (CFES) was established to promote forest-based healthcare in China. On 30 November 2017, the Forest Rehabilitation and Recreation Committee (FRRC) of the China National Forest Farm Association (CNFFA) was founded. On 1 April 2018, the Forest Therapy Committee (FTC) of the China Forestry Society (CFS) was established. Professional organizations will spread and popularize the concept of forest therapy from multiple angles and provide professional services for theoretical studies, industry development, and technological exchange. 

In addition, a number of centers focused on people’s physical and psychological health were also established nationwide. In March 2019, SFGA, MCA, NHC, and SATCM jointly published the Opinions on Promoting the Development of Forest rehabilitation and recreation Industry. This report states that 300 (1200) national forest rehabilitation and recreation centers will be built by 2022 (by 2035) to provide quality forest therapy services at different levels and satisfying people’s increased need for a healthy lifestyle. The “Forest Rehabilitation and Recreation Center Contest” was organized by CFIA four times to data, and 374 centers were awarded for a national pilot program. Meanwhile Beijing, Hunan, Sichuan, Guizhou, Zhejiang, and Shanxi have built several provincial and municipal forest therapy centers. 

## 3. Challenges

In our opinion, forest therapy is an ancient therapy based on the relationship between humans and nature. The ancient Chinese belief of “following the law of nature” and “harmony between humans and nature” and the western belief of “biophilia hypothesis”, “attention restoration theory”, and “stress recovery theory” are based on the simple ecological concept that forests can provide the health factors to humans. However, human history shows that there are contradictions between our beliefs and actions. Mentally, we are longing for nature, but our actions have been destroying and modifying nature. Today, we are developing forest therapy in China, propose an action plan, and aim to identify the correlation between human health and forests. We want to take advantage of the forest’s health functions and to improve human health. Although we have made some progress, we face many challenges. 

### 3.1. Weak Basic Research

To date, evidence-based medical research on forest therapy has been relatively rare, and little scientific evidence has been obtained; basic research is weak. However, the forest therapy industry has developed rapidly in recent years, and a nationwide movement has resulted in the establishment of forest therapy centers and the development of forest-based health activities. If there is no answer to the core question “What kind of forest environment or what activities can alleviate what kind of health problems”, forest therapy cannot be developed. Without the essential techniques, the development model will be reduced to a backward capacity. Therefore, we need to strengthen basic research, collect scientific evidence, and address the conflict between weak basic research and the rapid development of the industry. These actions are required so that the forest environment can be used to improve human health. 

### 3.2. Limitations of Experimental Design

During reviewing the literatures, all the experimental design of studies had been proven to be a powerful tool of exploring the relationship between nature and human health; however, the methods had some limitations due to different studies fields, experimental conditions, and theories. The sample size of studies is relatively small, which may be the limitation for the data statistics, then causing the results to be overestimated or underestimated [7]. Furthermore, individual differences such as underlying disease, educational background, physical activity and social interaction are also a difficult problem to address. Aspects of tree color, canopy shape, smell, and possibly touch feeling all need much more details to clarify the effect of forest therapy [38]. In addition, the effects of similar changes on climate and seasons is not clear [33]. More importantly, the relevant evidence of physiological response to different dominant tree species is insufficient to establish direct contact with them. Finally, based on current findings, a large-scale, multi-factor, and cohort study should be warranted.

### 3.3. Lack of Cooperation between Foresters and Doctors

Forest therapy is multi-disciplinary research that requires collaboration. In China, all forest therapy-related activities were led by the forest sector, which is in contrast to the activities in other countries. Since the forest provides the health function, people with forestry knowledge are required. However, medical professionals are needed to assess whether the forest environment benefits human health. Forest therapy also involves psychology, management, and other subjects. Currently, no effective cooperation has been established among the disciplines, and the latest research results in these disciplines have not been disseminated and integrated. To date, no interdisciplinary scientific concept has been established. Forest therapy centers often use the development and organizational methods of other industries. 

### 3.4. Inadequate Policy System

**Figure 2:**
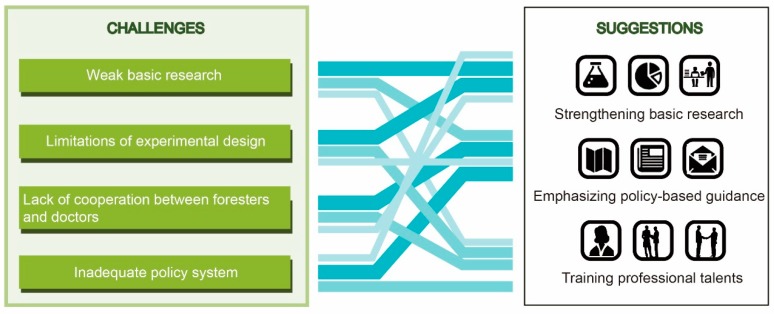
Challenges and suggestions for the development of forest therapy.

In 1989, Japan promulgated special measures for increasing the function of forestry health care, defining the health function of forest in the form of a special law. The legislation strongly promoted the development of forest therapy in Japan. At present, there is no definition of healthcare forest type in China. It will be conducive to the development of forest therapy if this type is added to the five existing forest types (shelter forest, timber forest, economic forest, firewood forest, and special forest) (Figure 2).

## 4. Suggestions for the Development of Forest Therapy

Due to urbanization, the health problems of people living in the urban jungle are unavoidable. People are longing for natural forests, but the human need for health far outweighs the services that forests can provide. In the long run, the conflict between the human need for health and the services that forests provide cannot be avoided. Therefore, forest therapy may be an effective solution to address this conflict. 

### 4.1. Strengthening Basic Research

Basic research is an important driver for innovation. Research institutions should conduct evidence-based medical research, collect experimental data, perform systematic forest therapy research, and develop a comprehensive basic research system. Professional research is usually time-consuming, but with scientific support, forest therapy can be developed rapidly. In addition, basic research requires a global vision. We need to obtain the latest research results from other countries to remain proactive. China has diverse forest types. We encourage research institutions to focus on different perspectives in research and on technological development and take a leading role in original research. In the future, both quantitative and qualitative studies on the health functions of forests should be enhanced, with particular focus on identifying a direct causative relationship between forests ecosystem services and the human health.

### 4.2. Emphasizing Policy-based Guidance

Many forest therapy-related policies have been issued on national and regional levels. Hunan, Sichuan, Guizhou and other provinces have witnessed the positive effects of policies in promoting the development of the forest therapy industry. What is needed right now is an improvement in the policies and the establishment of a government-led initiative in conjunction with private participation. With targeted policies and increased capital input, the whole society will take part in the industry, and the development of the industry would benefit from low-interest loans, tax cuts, and program-based financing. Meanwhile, the related costs of forest therapy activities should be committed to add in the national or regional medical insurance system, which should play a key role in enhancing overall sense of well-being and balance in life.

### 4.3. Training Professional Talents

As a multi-disciplinary, forest therapy involved various fields of forestry, medicine, psychology, management, and education. It includes extensive and complicated research fields and types of services. Multiple resources are needed; therefore, interdisciplinary collaboration, multi-disciplinary participation, and cultivation of professionals with multiple backgrounds are emphasized. Currently, several universities have established forest therapy-related departments to train professional talents, laying an important foundation for the development of forest therapy. We are training forest therapists and forest guides with good knowledge of the forest environment by using “online class + offline practice”. 

The Ancient Chinese Philosophy and Traditional Chinese Medicine supports the idea that forests are contributing to the balance between mind, body, and spirit. When being in or viewing forests, plants, flowers, urban green spaces, and natural wooden materials, people can obtain positive health benefits [17]. How to better apply the latest research results to solve practical problems is still a major problem faced by the majority of researchers and practitioners. On the basis of what has been achieved so far, the researchers need to actively explore new development concepts, and work with decision-making departments and forest therapy centers to maximize the health functions of forests, and let the general public feel the healthcare and well-being of forests. This will be the direction of our efforts in the future.

## Figures and Tables

**Table 1:** Characteristics of selected studies about forest therapy in China.

Study	Population	Sample	Setting	Aim & Design	Findings	Advantages	Limitations
Lyu [7]	Male college students from Sichuan Agricultural University were participated, none of the participants reported any physiological or psychiatric disorders in their personal histories, excluded subjects who smoked or alcoholic.	N = 60; ages from 19 to 24 years.	The bamboo forest site located near the city of Ya’an, the city site was located in the center of downtown in Chengdu city.	The subjects were randomly divided into four groups. The effects of bamboo forest therapy were explored by comparing the difference in the psycho-physiologic responses of participants before and after the test.	The bamboo forest therapy significantly increased natural killer cells activity, the number of natural killer cells and perforin-, granulysin-, and granzyme A/B-expressing cells and significantly decreased the corticosterone level in peripheral blood lymphocytes in the male participants.	This study is the first to research the benefits of bamboo forest therapy. An explanation of the mechanism underlying the interactions between the nervous, endocrine and immune systems is given.	The sample size is small; the subjects with illness is not included.
Tsao [16]	100 Staff members who live in the forest and 90 urban staff members who live in Taipei were recruited to determine the health effects on NK cells. 11 middle-aged volunteers were invited to investigate the health effects of a forest trip.	N = 211; mean ages 44.8 in the urban groups and 45.2 in the forest groups.	The forest site located in The Xitou Experimental Forest, the urban site located in Taipei city.	All participants were subjected to cardiovascular health and biochemical examinations and NK cell measurements. 11 middle-aged volunteers were invited to participate in a five-day/four-night forest trip to Xitou forest to investigate the health effects of a forest trip on NK cells and activating NK cells.	NK cells were higher in the forest group than in the urban group. The percentage of activating NK cells of the invited participants from Taipei increased significantly after the trip to Xitou forest.	On the basis of comparative experiments, the volunteers were added to participate in a forest trip to investigate the health effects of a forest trip.	The specific health effects of biogenic volatile organic compounds from tree leaves in forest environments are not investigated. The sample size of the forest trip group is small.
Mao [33]	Normal male university students were enrolled. None of the subjects reported any physiological or psychiatric disorders in their personal histories.	N = 20; age 20.79 ± 0.54 years.	The experiments were conducted in a broad-leaved evergreen forest in Wuchao Mountain, An urban area was used for comparison in Hangzhou city, Zhejiang Province.	The participants were randomly divided into two groups. One group was sent on a two-night trip to a broad-leaved evergreen forest, and the other was sent to a city area. Serum cytokine levels, the distribution of leukocyte subsets, and plasma endothelin-1 concentrations were measured before and after the experiment to evaluate the positive health effects of forest environments.	Mid- and short-time exposure to an evergreen broad-leaved forest could reduce oxidative stress and pro-inflammatory and serum cortisol levels.	The physiological and psychiatric indicator are together used to assess the health effects of forest environments.	The sample size is small. The factor of climate and air quality are not monitored at each experimental site.
Mao [34]	Elderly patients with diagnosed essential hypertension were recruited from Hangzhou.	N = 24; ages from 60 to 75 years.	The experiment was conducted in a broad-leaved evergreen forest in White Horse Mountain National Forest Park in Suichang County, an urban area in Hangzhou was used for comparison.	Two groups of participants were respectively sent to the forest or an urban control area for a 7-day trip to evaluate the effect of forest bathing on blood pressure. Blood pressure indicators, cardiovascular disease-related pathological factors and tumor necrosis factor were detected.	The results provided direct evidence that forest bathing has therapeutic effects on human hypertension and induces inhibition of the renin-angiotensin system and inflammation, and thus inspiring its preventive efficacy against cardiovascular disorders.	The physiological and psychiatric indicator are together used to assess the health effects of forest environments. The air quality in the two experimental sites is monitored.	The sample size is small. The age range of participants is limited. The factors of climate are not monitored.
Zhou [35]	The students majoring in forest ecology were recruited as volunteers from Guizhou University. They were informed to prohibit any of vigorous physical activity, smoking, and alcohol consumption before and throughout the whole experiment.	N = 43; 8 male and 35 female, ages from 19 to 23 years.	Qianlingshan Park and Xiaoche River Park were set as test site in Guiyang City, Guizhou Province.	To know about the urban forest therapy effect of anxiety alleviation with reference to the rural forest, the forest experience was separated by four sceneries. Participants were asked to complete questionnaires by self-evaluating specific anxiety change from 12 questions with scores from 1 to 10 at both entrance and exit of the parks.	University students were recommended to pay a short visit to the urban forest with partners if they felt anxious about personal affairs and felt necessary to talk with others. For general people’s visiting, urban forest trees can be controlled in diversity to some extent to look orderly and alleviate perceived anxiety.	This study find that forest bathing can alleviate perceived anxiety even in very detailed aspects, such as financial state, exam pass pressure, and love-affair relationship.	The sample size is small. The factors of forest and climate are not monitored. The physiological indicators are not used to evaluate. The control site is not set.
Wang [36]	Undergraduate and graduate students without heart disease or a diagnosis of irregular heart beat were participated from Tongji University.	N = 140; 50% male and 50% female, ages from 18 to 24 years.	N/A	This study explored the stress recovery effects of different videotaped scenes, using six urban parks and one urban roadway scene. Subjects were randomly assigned to watch one of the seven video scenes, with twenty subjects watching each scene.	Urban park scenes relieved stress and restored attention levels, whereas viewing urban roadways increased negative feelings. Outdoor scenes without people were more restorative than scenes depicting people.	The videotaped scenes are used as stimuli. Compared with direct experience of a site, this method has the advantage of controlling extraneous conditions.	Only one semi-enclosed scene is included, and the types of urban park is also limited. The physiological indicator are not used to evaluate.
Mao [37]	Elderly patients with Chronic Heart Failure (CHF) were recruited in Hangzhou city.	N = 43	Huangtan forest park located in Pan’an County was set as the forest site and an urban site located in the downtown area of Hangzhou that was set as the control, Zhejiang Province.	To further investigate the duration of the impact and the optimal frequency of forest bathing trips in patients with CHF, those subjects who had experienced the first forest bathing trip were recruited again after 4 weeks and randomly categorized into two groups, namely, the urban control group (city) and the forest bathing group (forest).	A steady decline in the brain natriuretic peptide levels, and an attenuated inflammatory response as well as oxidative stress. The additive benefits of twice forest bathing trips in elderly patients with CHF were demonstrated.	Additive benefits of twice forest bathing trips are demonstrated. This study provides supportive evidence that two 4-day forest bathing trips with a 4-week interval could offer additive benefits in elderly patients with CHF.	The sample size is small. The factors of forest and climate are not monitored. The age range of participants is limited.
Guan [38]	All participants were recruited from undergraduates in the major of urban horticulture from Jilin Agricultural University. They were informed to prohibit any of vigorous physical activity, smoking, and alcohol consumption before and throughout the whole experiment.	N = 69; 25 male and 44 female, ages from 19 to 22 years.	The study site locates at the Nanhu Park in Changchun City, Jilin Province.	This study aimed to evaluate the tree-species effect of forest bathing on perceived anxiety alleviation. The participants were recruited to visit urban forests dominated by birch, maple and oak trees, respectively.	The anxiety of the participants was reduced in the maple forest, the largest anxiety alleviation effects were observed in the birch forest, and female participants perceived more anxiety alleviation than male participants.	This study finds that urban forests have a tree-species-specific effect on anxiety allevia tion in university students.	The number of species, psycholog ical mechanism and physiological responses are limited. The control site is not set.
Tsao [39]	107 forest staff members (FSM) and 114 urban staff members (USM) to recruited to determine the long-term health effects of a forest environment.	N = 221; mean ages 43.2 in USM and 44.3 in FSM.	The FSM working in an experimental forest of National Taiwan University, Nantou County and the USM working in an urban environment in Taipei city.	To demonstrate the long-term health effects of living in a forest environment on subclinical cardiovascular diseases (CVDs) and health-related quality of life (HRQOL) compared with that in an urban environment. The detailed health examination and questionnaire assessment were investigated by the FSM and USM.	Levels of total cholesterol, low-density lipoprotein cholesterol, and fasting glucose in the USM group were significantly higher than those in the FSM group. Furthermore, a significantly higher intima-media thickness of the internal carotid artery was found in the USM group compared with that in the FSM group.	FSM working in an experimental forest and USM working in an urban environment is recruited. TheFSM group or USM group have worked in the forest or urban environment for more than 1 year.	The CV effects of changes in seasons have not been considered. The beneficial health factors of a forest environment have not been monitored and assessed.
Jia [40]	Elderly patients with chronic obstructive pulmonary disease (COPD) were enrolled in Hangzhou city, who had been without the acute exacerbation for at least 6 weeks.	N = 20	The experiment was conducted in a broad-leaved evergreen forest in White Horse Mountain National Forest Park in Suichang County, an urban area in Hangzhou was used for comparison.	This study aimed to evaluate the health effects of forest bathing trip on elderly patients with COPD. The patients were randomly divided into two groups. One group was sent to forest, and the other was sent to an urban area as control.	In the forest group, there was a significant decrease of perforin and granzyme B expressions, accompanied by decreased levels of pro-inflammatory cytokines and stress hormones. Meanwhile, the scores in the negative subscales of POMS decreased after forest bathing trip.	The physiological and psychiatric indicator are together used to assess the health effects of forest environments.	The sample size is small. The factors of forest and climate are not monitored.
Yu [41]	Middle-aged and elderly participants were recruited. 59 subjects (46.1%) reported chronic diseases including diabetes, hypertension, heart and other diseases.	N = 128; 85 females and 43 males, age from 45 to 86 (60.0 ± 7.44) years.	The experiment site was in the planted forest mainly containing *Cryptomeria japonica* and *Phyllostachys pubescensand*, the stand age ranged between 40 and 90 years old in Xitou Nature Education Area.	This study sought to understand the physiological and psychological effects of the short forest bathing program on middle-aged and elderly individuals. Physiological responses, pulse rate, systolic and diastolic blood pressure, heart rate variability (HRV), and psychological indices were measured before and after the program.	The short forest bathing program is a promising therapeutic method for enhancing heart rate and blood pressure functions as well as an effective psychological relaxation strategy for middle-aged and elderly individuals.	The physiological and psychiatric indicator are together used to assess the health effects of forest environments. The sample size is big.	The effects of socio-economic status, medication usage, habits and personality are not collected. The environmental factors and environmental conditions are not included as covariates.
Wang [42]	College students and social workers were selected as effective participants. All the subjects had normal vision or corrected normal vision, no mental disorder, no stress disorder, no abnormal organic disease, no brain trauma, and no endocrine diseases.	N = 96; 33 males and 63 females, age 24.03 ± 5.29 years.	N/A	To explore the effects of different types of forest environments for forest therapy, the study focused on forest resting environments. Seven representative forest resting environments found in field research in Beijing were used as independent variables and were shown to subjects by a virtual reality (VR) video.	This study found that all the seven different types of forest resting environments can produce stress relief effects to some extent. Different types of forest resting environments have different effects on relieving stress. The most natural environment does not have the most significant effect on stress relief. A water landscape has a positive effect on the relief of stress.	This study find that different types of forest resting environments have different effects on relieving stress.	Only seven types of forest resting environments are explored. The visual angle of the VR video was fixed. Only visual factors are focused.
